# Imaging single DNA molecules for high precision NIPT

**DOI:** 10.1038/s41598-018-22606-0

**Published:** 2018-03-14

**Authors:** Fredrik Dahl, Olle Ericsson, Olof Karlberg, Filip Karlsson, Mathias Howell, Fredrik Persson, Fredrik Roos, Johan Stenberg, Tarja Ahola, Ida Alftrén, Björn Andersson, Emelie Barkenäs, Birgit Brandner, Jenny Dahlberg, Sara Elfman, Magnus Eriksson, Per-Ola Forsgren, Niels Francois, Anna Gousseva, Faizan Hakamali, Åsa Janfalk-Carlsson, Henrik Johansson, Johanna Lundgren, Atefeh Mohsenchian, Linus Olausson, Simon Olofsson, Atif Qureshi, Björn Skarpås, Anna Sävneby, Eva Åström, Ove Öhman, Magnus Westgren, Helena Kopp-Kallner, Aino Fianu-Jonasson, Argyro Syngelaki, Kypros Nicolaides

**Affiliations:** 1 Vanadis Diagnostics, a PerkinElmer company, Stockholms Lan, Sweden; 2 Karolinska Institute, Solna, Sweden; 3 Danderyds Hospital Sweden, Danderyd, Sweden; 4 Harris Birthright Research Centre for Fetal Medicine, London, UK

## Abstract

Cell-free DNA analysis is becoming adopted for first line aneuploidy screening, however for most healthcare programs, cost and workflow complexity is limiting adoption of the test. We report a novel cost effective method, the Vanadis NIPT assay, designed for high precision digitally-enabled measurement of chromosomal aneuploidies in maternal plasma. Reducing NIPT assay complexity is achieved by using novel molecular probe technology that specifically label target chromosomes combined with a new readout format using a nanofilter to enrich single molecules for imaging and counting without DNA amplification, microarrays or sequencing. The primary objective of this study was to assess the Vanadis NIPT assay with respect to analytical precision and clinical feasibility. Analysis of reference DNA samples indicate that samples which are challenging to analyze with low fetal-fraction can be readily detected with a limit of detection determined at <2% fetal-fraction. In total of 286 clinical samples were analysed and 30 out of 30 pregnancies affected by trisomy 21 were classified correctly. This method has the potential to make cost effective NIPT more widely available with more women benefiting from superior detection and false positive rates.

## Introduction

Prenatal screening based on cell free DNA (cfDNA), also referred to as non-invasive prenatal testing (NIPT), has rapidly been adopted to become the standard follow-up procedure for patients classified as high risk using traditional combined first trimester screening (FTS)^1^. Comparing performance, FTS detects 85–90% of T21 cases with a false positive rate of 3–5% of screened patients, whilst cfDNA based tests detects 99.3% of T21 cases with a false positive rate of 0.1%^2–4^. Therefore NIPT, in its current implementation, has reduced the number of invasive tests performed. However, the overall detection rate of screening programs has not improved since the missed cases are among patients classified as low risk in the first trimester screen and therefore not offered cfDNA screening. To benefit from NIPT performance and improve the overall detection rate of aneuploidy screening programs, the cfDNA based screening needs to be provided to more women that opt-in for prenatal screening as a first line or contingent screening model. To enable this, cfDNA based screening needs to become more cost effective and accessible for more laboratories.

The majority of available NIPT solutions are using the digital quantification power of advanced sequencing platforms^2^. These solutions are expensive and complicated for laboratories to establish and operate which in turn increase test costs and consequently limit accessibility of NIPT for pregnant women. Efforts to reduce the cost per sample for sequencing has led to introduction of targeted PCR assays upstream of the sequencing readout platform at the expense of a more complex sample preparation and increased overall no-call rate^5–8^. Other proof of concept NIPT solutions using digital PCR^9^, MLPA^10^ and methylation based analysis^11^ have been demonstrated as potential alternative cost effective NIPT methods. To our knowledge these solutions have not been widely adopted in clinical practice since clinical performance is not on par with existing solutions. To reduce both complexity and cost of cfDNA based aneuploidy screening, whilst maintaining high clinical performance, we are developing the Vanadis NIPT assay. This combines a high precision digitally-enabled quantification, with a streamlined cost effective readout format that can be readily automated and quality assured. The primary objective of this study was to assess the Vanadis NIPT assay with respect to analytical precision and clinical feasibility to correctly identify trisomy 21 pregnancies.

## Results

### Vanadis NIPT assay

A sequencing and PCR-free method was developed to enable a cost efficient and high precision measurement of chromosomal aneuploidies. Thereby, we were able to eliminate expensive sequencing, complicated sample preparation protocols, PCR bias and bioinformatics. The maximum precision of digitally-enabled assays is dictated by the number of molecules analysed. To enable quantification based on high molecular count numbers without PCR, two strategies were used. Firstly, probes were designed to capture and generate labelled rolling circle replication products (RCPs) from ~3500 loci on chromosome 21, thereby increasing the number of counts per chromosome equivalent in the sample. Secondly, an optically transparent nanofilter 96-well plate was developed to capture RCPs with high yield by mechanical filtering prior to imaging, thereby increasing the number of molecules analysed from the sample.

**Figure 1:**
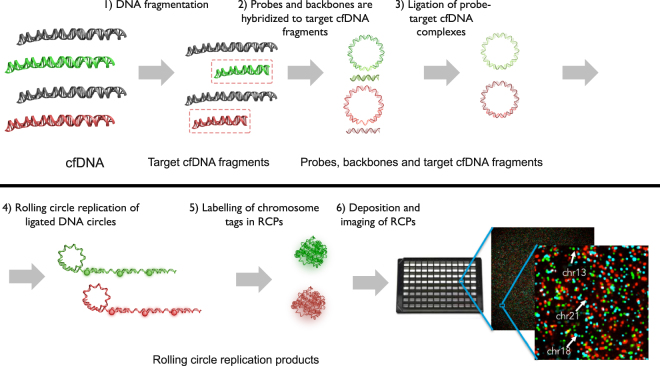
Vanadis NIPT assay. (1) Extracted cfDNA is first subjected to specific fragmentation using a restriction enzyme. The resulting target cfDNA fragments are similar in size and GC content and are derived from the chromosomes of interest. (2) Probes, designed to hybridize to the target cfDNA fragments to form circular DNA complexes, are mixed with the target cfDNA fragments, backbone oligos and DNA ligase. (3) By allowing the target cfDNA fragments to hybridize to the probe complex and DNA ligase to seal the nicks, covalently closed circles are generated that each includes a cfDNA target fragment and a corresponding chromosomal tag. All DNA that is not circularized is removed with exonucleases. (4) The DNA circles are copied about 1000 times by rolling-circle-amplification (RCA) to generate one rolling circle replication product (RCP), a single stranded concatemer amplification product. (5) The RCPs self-assemble to submicron-sized DNA objects. Because each RCP includes copies of a chromosomal tag it can be recognized by a corresponding fluorescently labeled oligonucleotide. (6) The labelled RCPs are then deposited to a 96-well nanofilter microplate. The microplate has a nanofilter membrane in the bottom to allow the RCPs to be captured on the plate bottom, while buffer and non-hybridized fluorophores are washed through the membrane. The deposited RCPs are finally imaged through the nanofilter using the Vanadis View imaging instrument.

The Vanadis NIPT assay (Fig. 1) is based on four consecutive enzymatic steps that specifically generate labelled RCPs from chromosomal DNA targets. The specificity of the DNA labelling approach eliminates the need for DNA sequencing and advanced bioinformatics data analysis. First, target chromosomes are digested into defined target cfDNA fragments using a restriction enzyme. Secondly, the digested target cfDNA fragments are mixed with a probe set where each probe carries a complementary sequence motif to the target cfDNA fragments of interest. The mixture also contains backbone oligonucleotides carrying a chromosome-specific sequence motif (“chromosomal tag”) used for subsequent labelling and identification. The probes are designed to specifically guide hybridization of target cfDNA fragments, thereby allowing subsequent DNA ligation of the target cfDNA fragments to the backbone oligo, such that a single stranded DNA circle can be formed. For this to occur, the selected cfDNA fragments need to hybridize perfectly to the probe and ligate both the 3´and 5´ends to the backbone. The chromosomal tags within the backbones are designed so that all target cfDNA fragments from one chromosome are ligated to identical backbones, enabling chromosome specific labelling. Following ligation, all remaining linear genomic DNA and unreacted probes are removed by exonucleases. Following clean up, primers are added and all DNA circles are reacted with a polymerase that copies the DNA circles to a clonal concatenated rolling circle replication product (RCP). Each original cfDNA target fragment generates one clonally amplified RCP which in solution collapses into a sub-micron sized DNA bundle. The RCPs are then labelled by adding fluorescent oligonucleotides complementary to the chromosomal tag sequences in the RCPs. All RCPs with the same chromosomal tag are labelled with identical fluorophores. After labelling, the reaction is added to a nanofilter 96-well plate. The solution passes through the filter while the RCPs are immobilized on the filter surface. The RCPs are washed and then immobilized on the filter using a silicone based optical clearing agent. The labelled RCPs are then imaged through the bottom of the plate using the Vanadis View^®^plate scanner for subsequent quantification.

### Estimating technical requirements for clinical performance

**Figure 2:**
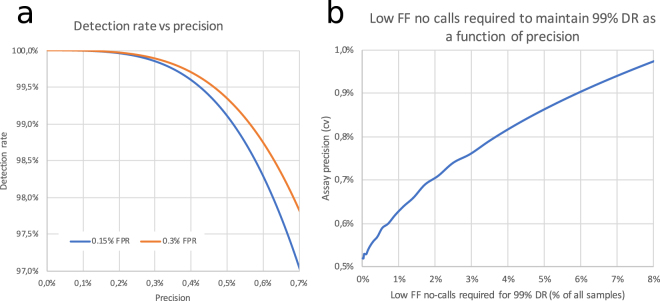
(**a**) Graph outlining the detection rate as a function of the assay precision for false positive rates 0.15% and 0.3%. (**b**) Graph outlining the fraction of samples that need to be eliminated to achieve 99% detection rate with 0.15% false positive rate at different measurement precisions.

To estimate the precision required to achieve a desired detection rate for trisomy 21 a model was created based on a well characterized data series published by Revello *et al*. in a cohort of 10 698 pregnancies from gestational weeks 10–14^7^. The key assumptions are that the average fetal fraction is ~11% for both normal and T21 pregnancies and follows a distribution where the square root of the fetal fraction is Norm(0.32, 0.07). For detection rate estimation at different assay precision a false positive rate of 0.15% and 0.3% are shown. To achieve a ≥99% detection rate with these parameters a coefficient of variation (cv) of around 0.5–0.6% or better precision is required (Fig. 2a). If the required precision cannot be achieved assays can either be designed with higher false positive rates (Fig. 2a) or samples with low fetal fraction can be eliminated. Using the same model Fig. 2b outlines what fraction of samples need to be eliminated when precision deteriorates to still achieve 99% detection rate.

**Figure 3:**
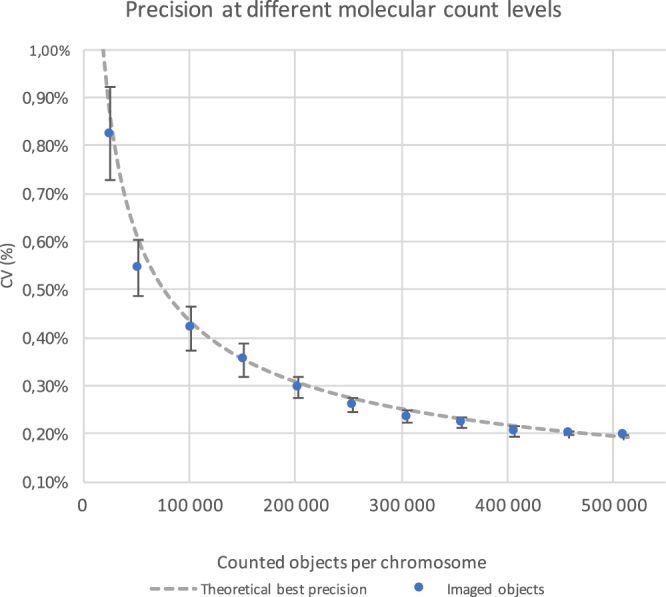
To evaluate the quantification precision of the Vanadis NIPT assay counting readout, a plate was analysed with identical input. The variation of the ratio-measurement is set by the number of objects quantified. When fewer objects are imaged the precision deteriorates according to the theoretically optimal measurement precision. Standard deviations are plotted from five randomized down samplings.

### Technical assay performance

The capture specificity of the probe set was evaluated by sequencing. For three probe sets, capturing 3500 loci at chromosomes 13, 18 and 21, ≥99.9% of the sequence reads aligned to the correct target loci in the genome. Extraction of cfDNA from plasma samples generated approximately 12 ng DNA and 600 000 counts per sample and chromosome. To evaluate the precision achieved by the Vanadis NIPT assay digitally-enabled counting readout, cell line samples reacted to labelled RCPs were pooled and split across one nanofilter plate and quantified. In average 508 842 objects per well and chromosome were counted and the normalized ratio across samples had a standard deviation of 0.2%, which corresponds well with the theoretically lowest variability possible at this count depth (Fig. 3), assuming Poisson distributed counts between samples by:

1 $$theorC{V}_{Count{s}_{affected}/Count{s}_{reference}}=\sqrt{\frac{1}{\langle Count{s}_{affected}\rangle }+\frac{1}{\langle Count{s}_{reference}\rangle }}$$

Down-sampling the data by successively removing images deteriorates the precision in accordance with the theoretical model (Fig. 3). Processing 24 identical reference plasma samples through cfDNA extraction, labelling and counting increased variation slightly to 0.29% indicating minimal contribution of the measurement variability by the DNA extraction and Vanadis NIPT assay.

**Figure 4:**
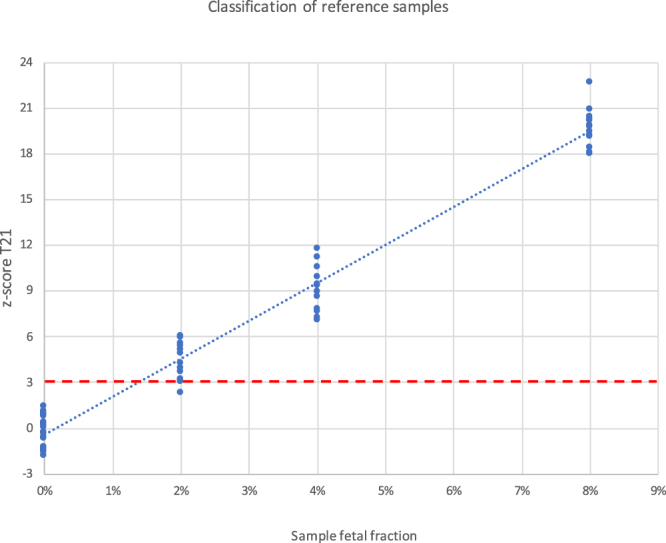
Reference samples from SeraCare with 0%, 2%, 4% and 8% T21 DNA were analysed. All samples with 4% and 8% T21 DNA, and all but one out of 14 samples with 2% T21 DNA are separated from normal with >3 standard deviations.

The ability to measure samples with low fetal fraction was evaluated using reference samples from SeraCare with predetermined amounts of trisomy DNA. Reference samples with 0%, 2%, 4% and 8% T21 were analysed. All samples with trisomy DNA with fetal fraction of 4% or higher were clearly identified >3 standard deviations from reference samples, and all but one of the 2% fetal fraction trisomy samples were >3 standard deviations from reference samples (Fig. 4). The measured assay precision was 0.2% of the normal samples.

### Clinical performance proof of concept

**Figure 5:**
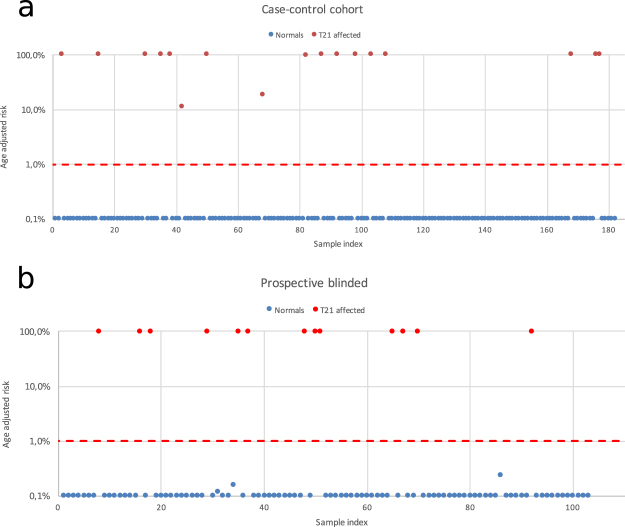
(**a**) 17 clinical samples from pregnancies with T21 were analysed among a set of 182 samples. All affected samples were identified correctly. (**b**) 104 prospectively collected samples were collected analysed blindly. 13 positive trisomy 21 pregnancies were classified correctly with no false positives.

To investigate detection of fetal chromosomal aneuploidies in clinical samples, analysis of T21 pregnancies was performed using a cohort of affected T21 pregnancies confirmed with invasive diagnostic procedures randomly distributed with samples from healthy pregnancies confirmed by outcome. Women carrying fetuses with trisomy 21 had an average gestational age of 16 weeks, average maternal age was 35 years and weight 66 kg. The average gestational week for the cohort of women with euploid pregnancies was 12, the average maternal age 31 years and average weight 66.0 kg. In total, 17 samples from affected T21 pregnancies were analysed blindly among 165 samples from healthy pregnant women. Using an age adjusted risk cut-off higher than 1%, all affected and normal samples were classified correctly (Fig. 5a).

To assess performance for analysis of samples collected prospectively in a clinical setting, samples were collected from pregnant women classified as high risk and subjected to invasive diagnostic procedures at Kings College in London. In total, blinded samples from 104 pregnant women were analysed for increased risk of trisomy 21. The average gestational age was 13 weeks and 4 days (+/−1 week and 6 days), average age 33 years and average weight was 70.5 kg. In total there were 13 positive trisomy 21 pregnancies which all were classified correctly using an age adjusted risk cut-off of 1%. No false positives were recorded and one sample failed due to insufficient DNA (Fig. 5b).

## Materials and Methods

### Study population

For the case-control study two sample sets were collected; confirmed trisomy 21 pregnancies samples were collected from pregnant women carrying one affected fetus, samples were collected in association with termination. One cohort of women with euploid singleton pregnancies were collected in association with first trimester screening after gestational week 9. In total 17 samples from pregnancies affected with trisomy 21 was collected and 165 samples from normal pregnancies. Aneuploidy status was confirmed for all patients by outcome at birth or invasive diagnostic procedures.

For the prospective high risk sample cohort plasma samples were collected prospectively before invasive testing from singleton pregnancies at week 11–22 classified as high risk for trisomy 21. All patients were examined by ultrasound to record the gestational age by measurement of the crown-rump length, to diagnose major fetal abnormalities and to measure NT thickness. Maternal serum levels of pregnancy associated plasma protein (PAPP)-A and free β-chorionic gonadotropin (hCG) were determined. Biophysical and biochemical biomarkers were combined to estimate a patient specific risk score.

All women provided informed consent to participate in the studies herein which were reviewed and approved by King’s College Hospital Ethics Committee and the ethical review board in Stockholm, Sweden (Regionala etikprövningsnämnden, Stockholm). All methods were performed in accordance with the relevant guidelines and regulations.

Samples with twin pregnancies, detection of other aneuploidies during invasive testing, failure to ship and process plasma within five days or if no follow-up or invasive test result available were excluded from the study.

### Plasma isolation

Plasma was isolated within five days from blood draw. An average of 9.5 mL whole blood was collected in Cell-free DNA BCT® blood collection tubes (Streck) via a double centrifugation protocol consisting of a first centrifugation step at 1342 × g for 30 minutes, transfer of the plasma fraction to a secondary tube and a second centrifugation step at 2267 × g for 20 minutes. Plasma was stored at −80 C until further processing.

### Detailed Vanadis NIPT assay protocol

cfDNA was extracted from 3 ml of plasma via a bead based protocol. 50 µl of each cfDNA eluate was transferred to a microtiter plate and digested in 60 µL reaction containing 5 U MseI (New England Biolabs), 50 mM NaCl, 10 mM MgCl_2_ and 10 mM Tris-HCl pH 8.0. The reaction was incubated at 37 °C for 30 minutes, followed by 65 °C for 20 minutes. Following restriction endonuclease digestion, a 70 µl hybridization and ligation reaction was added to produce a reaction mixture containing 5 pM per locus-specific probe, 508 nM per backbone (IDT), 80 U of Taq DNA Ligase (Qiagen), 1 mM NAD^+^ (New England Biolabs), 100 mM NaCl, 15 mM MgCl_2_, 10 mM Tris-HCl pH 8 and 0.11% w/v Tween20. The reaction was incubated at 95 °C for 5 minutes, followed by 10 hours at 56 °C. Following hybridization and ligation, 39 µl of an exonuclease master mix consisting of 20 U Exonuclease I (New England Biolabs), 5 U Lambda exonuclease (New England Biolabs), 100 U Exonuclease III (New England Biolabs), 10 U Uracil dehydrogenase (New England Biolabs) and 28% Tween20 was added to each reaction. The mixture was incubated at 37 °C for 1 hour, followed by 80 °C for 20 minutes. Rolling circle amplification (RCA) was performed by adding 11.2 µl RCA master mix consisting of 1.07 µM of a RCA primer oligonucleotide (IDT) designed to be complementary to a sequence present in the backbone region of each DNA circle, 5.4 mM dNTP solution mix (New England Biolabs) and 20 U Phi29 DNA polymerase (New England Biolabs) to each reaction following exonuclease treatment. The RCA reaction was incubated at 37 °C for 1 hour, followed by 65 °C for 10 minutes. Following RCA a 12 µl labeling master mix was added to each reaction consisting of 60 nM of each fluorescent labeling oligonucleotide complementary to a chromosomal tag in the backbone, 12 × SSC buffer and 0.6% Tween20. The reaction was then incubated at 45 °C for 1 hour.

Each reaction was then filtered through a well of a nanofilter detection plate (PerkinElmer) fixing the labeled RCA objects to the upper membrane surface due to the small pore size of the nanofilter. The membrane was washed twice in 0.5 × SSC and the detection plate was moved to a blotting membrane to remove residual liquid. After drying the detection plate, an optical clearing agent (PerkinElmer) was added to each well and allowed to cure for 10 minutes. The plates were imaged and analyzed using a Vanadis View^TM^ microplate scanner (PerkinElmer).

### Image and Data analysis

Once per run, the crosstalk between spectrally neighboring channels was estimated after alignment of the channel pairs. The crosstalk is subsequently subtracted from the images prior to further analysis. An in-house developed filter identifies and masks out large bright regions from the images in all channels. After masking the images, the fluorescent RCA objects were enhanced using an atrous wavelet filter. For each reaction and spectral channel a threshold, based on the sum of class variances with inclusion of class discrepancy, was determined to separate the RCA objects from the image background. To separate nearby objects, local maxima with 8 connectivity within the image regions above the threshold was segmented into separate objects.

Both samples and images were subjected to quality control. Images with high tilt in the focal plane, too many detected objects or with outliers in chromosome ratio as compared to the rest of that sample were rejected from further analysis. Samples with too high variance between the image chromosome ratios and with a too few detected objects were rejected from further analysis.

The ratio between the chromosomes, represented by the spectral channels, were affected both by slight differences in specificity of the probe sets against different chromosomes and at high RCA object concentrations, and by the properties of the spectral detection channel. Therefore, a normalized ratio between the two chromosomes was determined by a robust quadratic regression. This regression uses all available reactions (samples and references), including the positive references in non-affected chromosome pairs.

The z-score calculations were performed using a standard deviation determined by an iterative approach using MAD (median absolute deviation) as a consistent estimator for standard deviation.

Age related prior risk/prevalence from Mai *et al*. was transformed to odds which was combined with the likelihood ratio from the test^12^. Subsequently the post-test odds were converted to a post-test risk and capped at 99% and 0.1% respectively. The distribution of positive T21 sample ratios used in the likelihood ratio were based on a fetal fraction distribution where the square root of the fetal fraction is described by a normal distribution N(0.33234, 0.068751) and is based on 10463 samples from Revello *et al*.^7^.

### Probe design

The Vanadis NIPT assay relies on incorporating target cfDNA fragments into DNA circles. Circularization of cfDNA target fragments generated by MseI digestion of the cfDNA was enabled by addition of the Vanadis NIPT probe set consisting of approximately 12 000 locus specific probe oligonucleotides which each contains a middle region complementary to a selected MseI digested target cfDNA fragment. Target cfDNA fragments were selected to be unique to the chromosomes of interest, to have uniform length and melting temperature and to not contain any known polymorphic bases.

**Figure 6:**
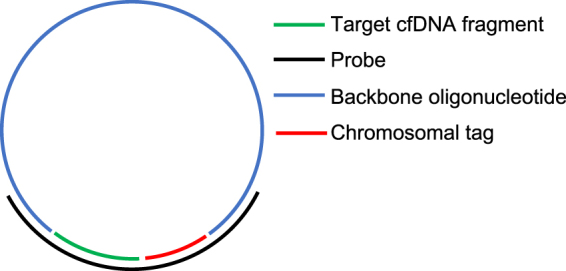
Schematic of Vanadis NIPT probe design. Correct hybridization of backbone oligonucleotide and MseI digested target cfDNA fragment generates a DNA circle, following closing of nicks by a DNA ligase. Each DNA circle contains sequence information of the incorporated target cfDNA fragment as well as a chromosomal tag enabling readout following fluorescent labeling of the chromosomal tag.

Additionally, the middle region of each probe contains a sequence complementary to a chromosomal tag, which is used to label RCPs and convey information about the chromosomal origin of the target cfDNA fragment. Furthermore, the outer regions of each probe was designed to be complementary to a backbone oligonucleotide, the outer regions flanking the middle region, thereby allowing the probe to guide the formation of a complete DNA circle containing a target cfDNA fragment, a chromosomal tag and a backbone oligonucleotide (Fig. 6).

## Discussion

In this study we could show that the Vanadis NIPT assay has the ability to achieve very high precision measurements for aneuploidy in clinical as well as standardized test samples. In order to improve the detection rate of aneuploidy screening programs through non-invasive testing, the cfDNA based tests need to be provided to more women that opt-in for prenatal screening. To achieve a wider adoption of NIPT in screening programs, less complex and lower cost solutions are required that can be readily adopted by high throughput screening laboratories.

A strength of the technique is that by using highly specific chromosomal labelling the Vanadis NIPT assay does not depend on advanced genetic readout systems like sequencing or microarrays thereby reducing assay cost and complexity. In addition, Interrogating ~3500 loci across each chromosome in combination with a nanofilter to increase molecular counting yield, eliminates the need for PCR. This has several advantages such as removing the risk for PCR contamination and reduction of assay variability contributed by DNA amplification. Counting a high number of molecules and avoiding PCR provides a high precision that enables accurate measurement of samples with low fetal fraction. Platforms that have adopted measurements of targeted PCR-based amplicons for NIPT typically eliminate 3–5% of patient samples from analysis since low fetal fraction samples cannot be measured reliably^5,13^. One contributing factor is likely the increased variability introduced by PCR. Eliminating samples with low fetal fraction reduces the false negative rate at the cost of increased no-call rate. Low-fetal fraction no-calls are challenging to resolve and several studies have shown that ~50% of these patients remain without a NIPT result even after being offered a second NIPT analysis due to repeat failures and poor patient compliance to submit samples for repeat testing^5,7^. In a clinical setting where NIPT is offered as a first line screen there would be T21 pregnancies in this group at high risk of remaining undetected. Our data show correct classification of reference DNA samples with 4% fetal fraction. Assay precision on plasma samples is estimated to ~0.3%, and using the model presented this would result in a detection rate for T21 of >99% with a 0.15% false positive rate. Increasing precision further provides a diminishing return in detection rate.

In the current study the probes were designed to analyze chromosome 21. The Vanadis NIPT assay however, is not limited to this chromosome, and can readily be expanded to analyze additional targets with up to four dyes, such as chromosome 13, 18, X, Y and microdeletions. Probes for additional chromosomes or microdeletions can be included in the same probe set, and with minor changes to the existing protocol, be imaged in separate wells or in the same well using combinatorial fluorescent labelling. In addition to prenatal aneuploidies there might also be an opportunity to adapt the assay to chromosomal loci with relevance for cancer screening and diagnostics.

This study outlines a novel NIPT assay with very high precision complemented with proof of principle clinical data classifying in total 30 T21 cases correctly. Additional and larger studies are required to demonstrate the application and performance of the Vanadis NIPT assay in a prospectively collected population cohort for screening trisomy 21 and additional chromosomes and microdeletions. By enabling high precision measurement, the presented approach have the potential to provide a high detection rate combined with a low no-call rate in a cost effective format. The assay is compatible with a standard 96 well plate workflow and automated imaging for data acquisition. This opens up for development of an automated system for aneuploidy screening that could be adopted by any laboratory. Availability of a cost effective, automated high-performance system is vital for reliable cfDNA prenatal testing to become an available and affordable option for all pregnant women.
